# Extracellular vesicles play a central role in linking podocyte injury to mesangial activation in glomerular disease

**DOI:** 10.7150/thno.110034

**Published:** 2025-04-09

**Authors:** Zhao Liu, Xi Liu, Yi Zhou, Xin Wen, Jie Xu, Meizhi He, Jiongcheng Chen, Nan Jia, Youhua Liu

**Affiliations:** 1 State Key Laboratory of Multi-organ Injury Prevention and Treatment, Division of Nephrology, Nanfang Hospital, Southern Medical University, Guangzhou, China.; 2 National Clinical Research Center of Kidney Disease, Guangdong Provincial Institute of Nephrology, Guangzhou, China.

**Keywords:** Glomerulosclerosis, podocyte-mesangial communication, extracellular vesicles, Shh, proteinuria

## Abstract

**Background:** Podocyte injury leading to proteinuria is the primary feature of a vast majority of glomerular diseases, while mesangial cell activation is the hallmark of glomerulosclerosis. Whether and how these two events are connected remains elusive. In this study, we investigated the role of extracellular vesicles (EVs) in linking podocyte injury to mesangial activation in glomerular disease.

**Methods:** EVs were characterized by nanoparticle tracking analysis and electron microscopy. Differentially expressed proteins from podocyte-derived EVs were analyzed by protein microarray. The role and mechanism by which EVs-packaged sonic hedgehog (Shh) mediates mesangial cell activation were investigated *in vitro* and *in vivo*.

**Results:** An increased production of EVs in mouse podocytes (MPC5) was observed after injury induced by angiotensin II (Ang II). Shh and N-Shh were identified as major constituents of the proteins encapsulated in EVs isolated from Ang II-treated MPC5 cells (Ang II-EVs). In vitro, Ang II-EVs induced the activation and proliferation of rat mesangial cells (HBZY-1), whereas inhibition of EV secretion with dimethyl amiloride, depletion of EVs from conditioned media or knockdown of Shh expression abolished the ability of Ang II-EVs to induce HBZY-1 activation. *In vivo*, intravenous injection of Ang II-EVs exacerbated glomerulosclerosis, which was negated by hedgehog inhibitor. Furthermore, blocking EV secretion also ameliorated glomerulosclerosis in mouse model of glomerular disease.

**Conclusions:** These findings suggest that podocyte injury can cause mesangial cell activation and glomerulosclerosis by releasing Shh-enriched EVs. Therefore, strategies targeting EVs may be a novel way to ameliorate proteinuric kidney disease.

## Introduction

Glomerular diseases are one of the most common causes of chronic kidney disease (CKD) leading to end-stage kidney failure worldwide 1, 2. The predominant pathological features of many glomerular diseases are characterized by proteinuria and glomerulosclerosis, which are primarily caused by podocyte injury and mesangial activation, respectively. Damage to podocytes, which are essential for maintaining the structural and functional integrity of the glomerulus 3, 4, results in protein leakage into the urine, a condition associated with poor renal outcomes 5, 6. Contrarywise, mesangial cell activation causes excessive production of extracellular matrix (ECM) leading to glomerular sclerotic lesions. Whether and/or how podocyte injury is linked to mesangial cell activation and the eventual development of glomerulosclerosis, however, remain elusive.

Both podocytes and mesangial cells are critical components of the glomerulus, and their interactions are believed to play a crucial role in the progression from proteinuria to glomerulosclerosis 7. Increasing evidence suggests that extracellular vesicles (EVs) mediate communication between various cell types in the kidney 8-10. EVs are lipid bilayer vesicles that capable of transferring RNA, lipids, proteins, and other materials between cells through secretion and endocytosis 11-13. These vesicles can influence not only neighboring cells but also distant ones, without requiring direct cell-to-cell contact. For instance, EVs secreted by injured kidney tubular epithelial cells can act on fibroblasts by carrying TGF-β1 mRNA, Shh ligand, osteopontin, and tumor necrosis factor-α-induced protein 8 (TNFAIP8), thereby inducing fibroblast proliferation, activation, ECM production, and resistance to apoptosis 14-17. Furthermore, EVs from injured tubular cells are enriched with miR-23a and monocyte chemoattractant protein-1 (MCP-1) mRNA, targeting macrophages and promoting their activation, thus exacerbating inflammation 9, 18, 19. Similarly, EVs derived from damaged podocytes may influence renal fibrosis by transporting miRNAs, p38 MAPK, and CD36, inducing apoptosis or directly triggering fibrotic responses in proximal tubular epithelial cells 9, 20. Unlike soluble secreted factors, EVs can transfer both ligands, receptors and signaling intermediators simultaneously to target cells, making EV-mediated signaling more efficient and robust 10. These findings prompted us to investigate whether EVs also play a role in mediating podocyte-mesangial communication, thereby linking podocyte injury to mesangial cell activation and glomerulosclerosis in glomerular diseases.

The hedgehog signaling is an evolutionarily conserved developmental signaling cascade that regulates various biological processes during embryogenesis, tissue homeostasis, and injury repair 21-23. Among the three hedgehog ligands, sonic hedgehog (Shh) is the most widely studied 24. A distinctive feature of Shh is that it is synthesized as a 45 kDa precursor, which undergoes autocatalytic cleavage to produce a biologically active 19 kDa N-terminal fragment (N-Shh), subsequently secreted into the extracellular space 25, 26. The N-Shh carries dual lipid modifications, including a cholesterol moiety at the C-terminus and a palmitoyl group at the N-terminus, which make it less soluble but more capable of tethering with exosomal membranes 25, 27. However, whether EVs are involved in mediating podocyte-mesangial communication is largely unknown.

In this study, we demonstrate that Shh is upregulated in injured podocytes, packaged into podocyte-derived EVs and transferred to mesangial cells. We show that EVs-encapsulated Shh signaling is a key mechanism linking podocyte injury to mesangial cell activation, matrix deposition, and glomerulosclerosis. Our findings reveal a novel mechanism connecting proteinuria to glomerulosclerosis and suggest potential strategies for therapeutic intervention of glomerular diseases.

## Materials and Methods

### Animal model

Male BALB/c mice weighing 22-24g were purchased from Vital River Laboratory (Beijing, China) and housed in the standard environment with regular light/dark cycles and free access to water and chow diet. Mouse model of proteinuria/glomerulosclerosis was established by infusion of angiotensin II (Ang II) (4006473; Bachem) and intravenous injection of Adriamycin (ADR) (Sigma-Aldrich). This combined Ang II/ADR model developed robust proteinuria and glomerulosclerosis in a relatively short period of time. For Ang II infusion, osmotic pumps (model 2006, Alzet) were placed into the subcutaneous space of anesthetized mice through a 0.5-cm incision in the back of the neck, and Ang II were infused with the dose of 1.5 mg/kg body weight per day. After 14 days, ADR were then injected intravenously via tail vein at the dose of 8 mg/kg body weight. Mice were euthanized at 5 weeks after Ang II infusion, and the kidney tissues were collected for subsequent analysis.

For studying the effects of EVs, mice were divided into five groups, and the experimental design was shown in the relevant figures. EVs were collected from podocytes treated with or without Ang II, quantified by using the micro bicinchoninic acid (BCA) protein assay and injected intravenously (1 mg per mouse per time point, once per two days). To inhibit the secretion of EVs, we used a pharmacological approach by daily intraperitoneal injections of dimethyl amiloride (DMA) (Sigma-Aldrich) at the dose of 20 mg/kg body weight in 0.9% saline. For blocking the effect of Shh, daily intraperitoneal injections of cyclopamine (CPN) (Sigma, St. Louis, MO) at the dose of 5 mg/kg body weight were performed.

All animal studies were performed according to the National Institutes of Health guideline for the care and use of laboratory animals and approved by the Animal Experimentation Committee at the Nanfang Hospital, Southern Medical University.

### Determination of albuminuria

Urinary albumin was measured by using a mouse Albumin ELISA Quantitation Kit according to the manufacturer's protocol (Bethyl Laboratories Inc).

### Cell culture and treatment

The conditionally immortalized mouse podocyte cell line (MPC5) was originally provided by Peter Mundel (Massachusetts General Hospital, Boston, MA), and rat glomerular mesangial cell line (HBZY-1) was obtained from the China Center for Type Culture Collection (CCTCC, Wuhan, China). To propagate podocytes, cells were cultured at 33°C in RPMI-1640 medium supplemented with 10% fetal bovine serum (FBS) and 10 units/ml mouse recombinant IFN-γ (R&D Systems, Minneapolis, MN) to enhance the expression of a thermosensitive T antigen. To induce differentiation, podocytes were grown under nonpermissive conditions at 37°C in the absence of IFN-γ. Differentiated MPC5 cells were then seeded onto six-well plates. After serum starvation overnight, Ang II was added to the serum-free medium at different concentrations for various periods of time as indicated. For some studies, MPC5 cells were treated with 10 nM Ang II for 6 h, and then Ang II was removed, followed by incubating in serum-free medium. In some experiments, MPC5 cells were pretreated with 100 μg/ml DMA, or transiently transfected with Shh siRNA using Lipofectiamine 2000 (Life Technologies, Grand Island, NY). The conditioned media were collected and subjected to EVs isolation. After serum starvation for 16 h, HBZY-1 cells were treated with MPC5 conditioned media or EVs (50 μg/mL) isolated from MPC5 cells. For some experiments, HBZY-1 cells were pretreated for 1 h with 10 µM CPN or 10 µM prochlorperazine (PCZ). Cells were then collected and subjected to various analyses.

### EV isolation

EVs were isolated from conditioned media by differential centrifugation. In brief, the initial spins were performed at 300 g for 5 min, 2000 g for 20 min, and 10,000 g for 30 min to remove cells and debris, the supernatant was then ultra-centrifuged at 110,000 g for 80 min. The pellets were suspended in ice-cold phosphate-buffered saline (PBS) to remove contaminating protein, then collected by ultra-centrifuged at 110,000 g for 1 h. All steps were performed at 4°C. The final pellets were resuspended in ice-cold PBS, and then used for tail vein injection to mice or treatment with HBZY-1 cells in serum-free medium for 24 h. Some EVs were also used for Western blot analyses. EVs were quantified using the micro bicinchoninic acid (BCA) protein assay. For in vitro studies, HBZY-1 cells were treated with 50 μg/mL EVs. For tail vein injection to mice, 1 mg of EVs was used in one mouse per injection. For ELISA-based protein array and some Western blot analyses for EV proteins, the EVs derived from equal numbers of podocytes (1×10⁷ cells) were used.

### Urinary EVs from CKD patients

Human urine samples from healthy subjects and CKD patients were obtained from the Division of Nephrology at the Nanfang Hospital, Southern Medical University, with written informed consent from the patients. All urine samples were measured for creatinine levels. After adjusting all samples to equivalent creatinine levels through dilution, EVs were isolated from the adjusted urine samples. Research involving human samples was approved by the Human Subject Ethics Committee of the Nanfang Hospital, Sothern Medical University.

### Nanoparticle tracking analysis (NTA)

The hydrodynamic size and concentration of purified EVs were measured by using Zeta View PMX 110 (Particle Metrix, Meerbusch, Germany) and analyzed by using corresponding software Zeta View 8.04.02.

### Transmission electron microscopy

For transmission electron microscopy (TEM), the pelleted EVs isolated from Ang II-treated MPC5 cells or small pieces of kidney tissue from Ang II/ADR mice were placed in 2.5% glutaraldehyde in PBS buffer and fixed. Samples were rinsed and post-fixed in 1% osmium tetroxide, then dehydrated in increasing concentrations of ethanol and infiltrated with increasing concentrations of epoxy resin mixed with propylene oxide. The samples were embedded in resin and cut into ultrathin sections with a diamond knife. The sections were then placed on copper mesh grids and stained with the heavy metal uranyl acetate for contrast. Samples were observed by a Tecnai transmission electron microscope at 120 kV (Thermo Fisher Scientific, Hillsboro, OR).

### Protein array

Mouse cytokine antibody arrays (G series 2000) were purchased from RayBiotech (Norcross, GA). EVs collected from MPC5 cells with or without Ang II treatment were processed according to the manufacturer's instructions.

### Fluorescent labeling of EVs

MPC5 cells were labeled with 1 mM Dil, a fluorescent lipophilic cationic indocarbocyanine dye (Life Technologies, Grand Island, NY) at the ratio of 200:1 in volume for 1 h and then washed three times with ice-cold PBS, followed by EVs isolation as described above to harvest. The Dil-labeled EVs were resuspended and incubated with HBZY-1 cells for 24 h or injected into mice via tail vein, then detected by immunofluorescence.

### Cell proliferation assay

Cell proliferation was assayed with a Cell Counting Kit-8 kit (CCK-8; Dojindo, Japan) according to the manufacturer's protocols. Briefly, 5x10^3^ HBZY-1 cells were seeded into each well of a 96-well culture plate at equal density and incubated overnight. For cell proliferation assay, the media were changed at 48 h, and 10 µl of CCK-8 reagent was added to each well and incubated for 2 h, plate was then read under the ELx800 Absorbance Reader (BioTek Instruments, Winooski, VT) at 450 nm.

The EdU incorporation assay was also performed to assess DNA synthesis. HBZY-1 cells were incubated with 0.2 mM EdU (Invitrogen) for 2 h, and then incorporated EdU was detected with mouse monoclonal anti-EdU antibody (B2531; Sigma-Aldrich). Stained samples were viewed under the confocal microscope (Leica SP8; Leica Microsystems, Buffalo Grove, IL).

### Western blot analysis

Kidney tissues, cells and EVs pellets were lysed in radioimmunoprecipitation (RIPA) assay buffer (Beyotime Biotechnology, Shanghai, China) and incubated at 4°C for 30 min. The protein concentration was measured by BCA protein assay 28. Equal amounts of protein were separated by 10% sodium dodecyl-sulfate-polyacrylamide gel electrophoresis (SDS-PAGE) and transferred onto polyvinylidene fluoride membranes (Millipore, Darmstadt, Germany). Membranes were blocked with 5% skim milk in Tris-buffered saline with 0.1% Tween 20 (TBST) buffer for 1 h and incubated with primary antibodies overnight at 4°C. After washing with TBST buffer, membranes were further incubated with appropriate horseradish peroxidase (HRP)-conjugated secondary antibodies at room temperature for 2 h. Protein bands were visualized with the HRP-enhanced chemiluminescence western blotting substrate. The primary antibodies and secondary antibodies used are listed in Supplementary Table S1.

### Histology and immunohistochemical staining

Formalin-fixed paraffin-embedded mouse kidney sections (3 µm) were prepared, dewaxed, washed in down-graded ethanol and water by a routine procedure. Periodic acid-Schiff (PAS) and Masson's trichrome staining (MTS) were performed using routine protocol 29.

For immunohistochemical staining, deparaffinized sections were incubated with 3% H_2_O_2_ solution to inhibit endogenous peroxidase activity. After blocking with 10% normal donkey serum, these slides were incubated with primary antibodies and secondary antibodies. Visualization was facilitated by the AEC or DAB substrate solution (Vectorlabs). Haematoxylin was used for nuclear counterstaining. The primary antibodies and secondary antibodies used are listed in Supplementary Table S1.

### Immunofluorescence staining

Kidney cryosections were fixed with 4% paraformalin for 15 min at room temperature. MPC5 or HBZY-1 cells cultured on coverslips were fixed with cold methanol: acetone (1:1) for 15 min at room temperature, followed by blocking with 0.3% Triton X-100 (Sigma-Aldrich) for 15 min and 10% normal donkey serum in PBS for 1 h at room temperature. Slides were then incubated with primary antibodies at 4°C overnight. After washing, the slides were then incubated with Cy3 or Cy2-conjugated donkey anti-mouse or donkey anti-rabbit IgG (Jackson Immuno-Research Laboratories, West Grove, PA) at room temperature for 2 h. Nuclei were stained with DAPI (Sigma-Aldrich) according to manufacturer's instruction. The slides were then observed under the confocal microscope (Leica SP8; Leica Microsystems, Buffalo Grove, IL).

### Quantifications of staining

Immunohistochemical, immunofluorescence was quantified at high-powered (×400, ×630) fields from randomly selected 3 fields each section 30. Quantification of positive staining was assessed by two researchers who were blinded through Image Pro Plus software.

### Statistical analysis

All data examined were expressed as mean ± standard error of mean (SEM). Statistical analyses of the data were performed using SPSS 21.0 (SPSS Inc, Chicago, IL). Comparisons between groups were made using one-way ANOVA, followed by Student-Newman-Kuels test or Dunnett's T3 procedure. *P* < 0.05 was considered significant.

## Results

### Podocyte injury is associated with increased production of EVs

**Figure 1:**
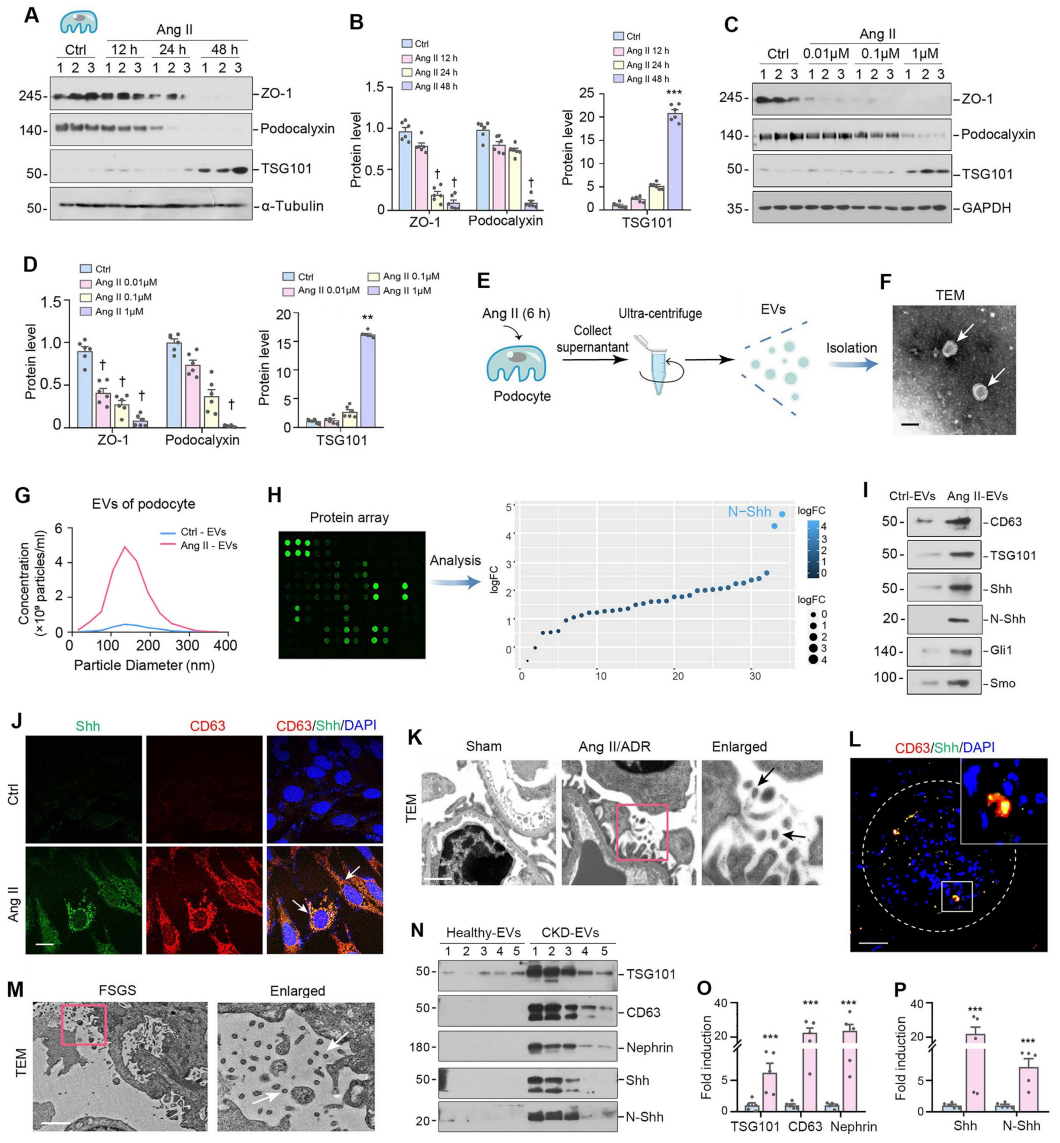
** Podocyte injury is associated with increased production of extracellular vesicles (EVs).** (**A-B**) Western blot analyses demonstrate protein expression of ZO-1, podocalyxin and tumor susceptibility gene 101 (TSG101) in mouse podocyte cells (MPC5) at different time points after Ang II treatment. Representative Western blot (A) and quantitative data (B) are presented. Numbers (1 to 3) indicate each individual culture plate in a given group. ^†^*P* < 0.05, ^***^P < 0.001versus control (n = 6). (**C-D**) Western blotting (C) and quantitative data show protein expression of ZO-1, podocalyxin and TSG101 (D) in MPC5 cells treated with different concentrations of Ang II. Numbers (1 to 3) indicate each individual culture plate in a given group. ^†^*P* < 0.05, ^**^P < 0.01 versus control (n = 6). (**E**) Diagram shows the experimental design of EVs isolated from MPC5 cells treated with Ang II (Ang II-EVs). (**F**) Transmission electron microscopy (TEM) shows the EVs isolated from conditioned media of MPC5 cells. Scale bar, 200 nm. (**G**) Average size distribution and concentration of Ctrl-EVs and Ang II-EVs were detected by nanoparticle tracking analysis (NTA), respectively. (**H**) Diagram shows the experimental design of the ELISA-based protein arrays to identify the different composition of EVs isolated from MPC5 cells treated with or without Ang II (Ang II-EV or Ctrl-EV). N-Shh was the most increased protein in Ang II-EV, compared with Ctrl-EV. (**I**) Representative Western blotting shows the presence of CD63, TSG101, as well as Shh, N-Shh, Gli1 and Smo in the EVs isolated from the Ang II-CM. (**J**) Double immunofluorescence staining demonstrates co-localization of Shh (Green) and CD63 (Red) in MPC5 after Ang II treatment. Arrows indicate positive staining. Scale bar, 10 µm. (**K**) Representative transmission electron microscopy (TEM) shows EVs in the interstitial spaces between podocytes and mesangial area of Ang II/ADR-treated glomerulosclerotic mice. Boxed area is enlarged. Arrows indicate EVs. Scale bar, 200 nm. (**L**) Double immunofluorescence staining indicates the generation of EVs in the glomeruli of diseased kidney from FSGS patient. Kidney sections were co-stained for CD63 (Red) and Shh (green). White broken line highlights the boundary of glomeruli. Boxed area is enlarged. Scale bar, 20 µm. (**M**) Transmission electron microscopy (TEM) shows extracellular vehicles (EVs) in the spaces between podocytes and mesangial cells of FSGS patient's renal tissue. Boxed area is enlarged. Arrows indicate EVs. Scale bar, 1 µm. (**N-P**) Western blotting (N) and quantitative data (O, P) show protein expression of TSG101, CD63, nephrin, Shh and N-Shh (D) in urinary EVs isolated from CKD patients. Numbers (1 to 5) indicate urinary EV samples from each individual in a given group. ^***^P < 0.001 versus control (n = 5).

To simulate podocyte damage in glomerular diseases, we incubated mouse podocytes (MPC5) with different doses of Ang II for varying periods of time. As expected, Ang II inhibited the expression of ZO-1 and podocalyxin, as demonstrated by Western blotting analysis (Figure 1A-D), indicating that Ang II can cause podocyte damage. Interestingly, Ang II also induced the expression of tumor susceptibility gene 101 (TSG101), a marker for EVs (Figure 1A-D). We then isolated EVs from the conditioned medium of cultured MPC5 cells that were treated with or without Ang II (Figure 1E). The characterization of EVs using transmission electron microscopy (TEM) and nanoparticle tracking analysis (NTA) is presented in Figure 1F and 1G, respectively.

To gain insight into the components responsible for the EVs-mediated biology, we used an ELISA-based protein array to characterize the proteins encapsulated in podocyte-derived EVs. To this end, we isolated EVs from the conditioned media of cultured MPC5 cells treated with or without Ang II, and measured EV protein levels using an ELISA protein array. Figure 1H shows that N-Shh was the most predominant protein identified in the EVs from damaged podocytes. Moreover, we confirmed by Western blotting that the EVs obtained from MPC5 cells treated with Ang II (Ang II-EVs) contained both Shh and its active form, the N-terminal fragment (N-Shh). Notably, we also observed that smoothened (Smo) and Gli1 were also present in Ang II-EVs (Figure 1I), indicating that several downstream proteins involved in Shh signaling were simultaneously encapsulated in the EVs. Immunofluorescence staining demonstrated that Ang II induced Shh and another EV marker CD63 in MPC5 (Figure 1J). Importantly, co-localization of Shh and CD63 was observed in MPC5 after Ang II treatment (Figure 1J).

We then examined the ultrastructure of the glomeruli using TEM in mouse model of glomerular disease induced by Ang II plus ADR. As shown in Figure 1K, a large number of EVs in the space between podocyte and mesangial region in the diseased glomeruli of Ang II+ADR mice. A significant portion of these EVs had diameters between 30-160 nm in size. Furthermore, Shh protein was found to co-localize with CD63 in the diseased glomeruli of FSGS patients (Figure 1L), suggesting that Shh is a component encapsulated by EVs in humans as well. We also observed a significant amount of EVs within the space between podocytes and mesangial cells in FSGS patients (Figure 1M). Furthermore, we analyzed urinary EVs from healthy subjects and CKD patients by Western blot and demonstrated an elevated level of EV markers (TSG101, CD63), nephrin (podocyte marker), and Shh/N-Shh in the isolated urinary EVs from CKD patients (Figure 1N-P). The clinical data of these patients are presented in Supplementary Table S2. These findings suggest that podocyte injury is associated with an increased production of EVs, in which Shh is a major component.

### Inhibition of EV release from podocytes attenuates mesangial cell activation *in vitro*

**Figure 2:**
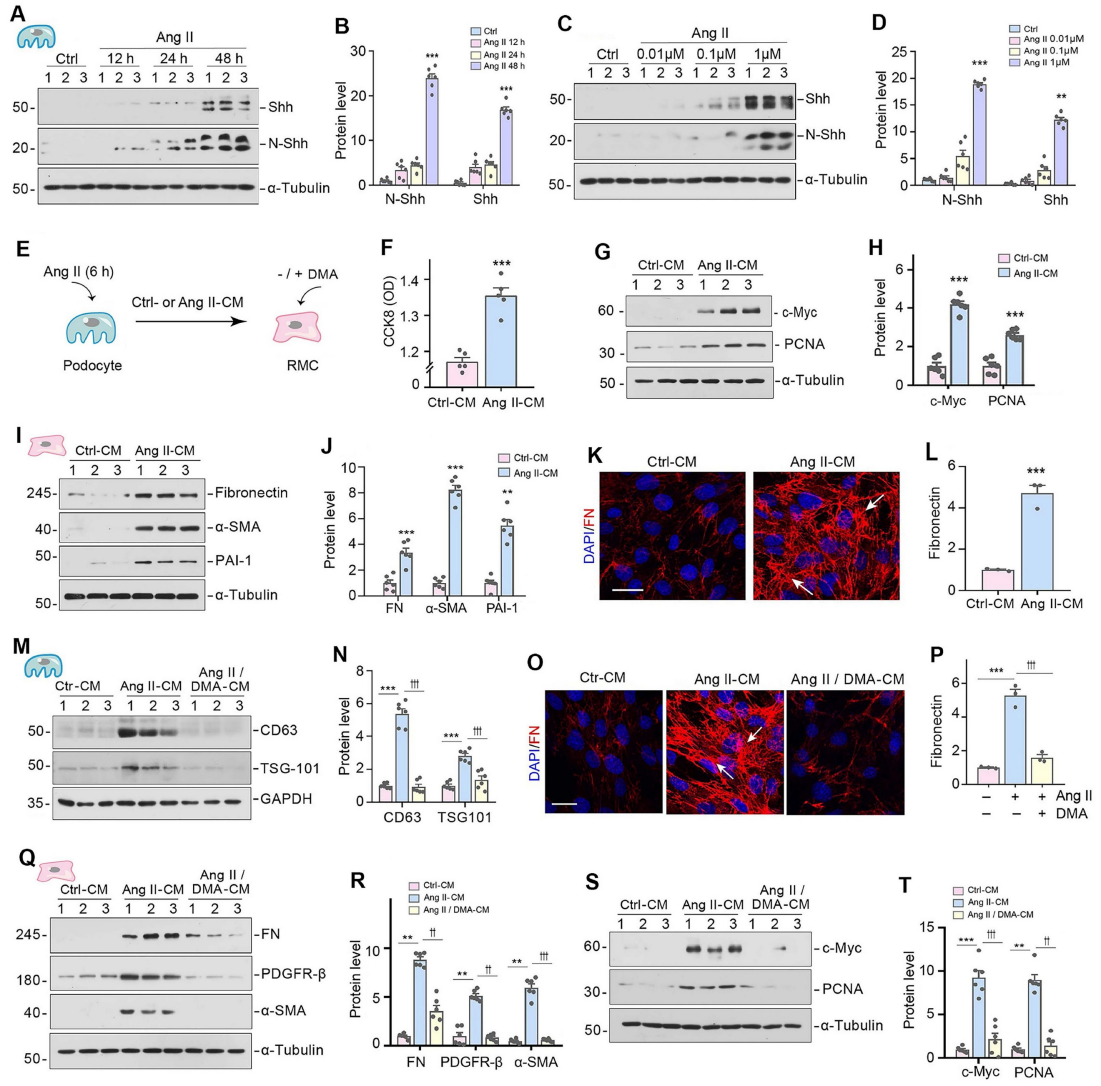
** Inhibition of EV release attenuates mesangial cell activation and proliferation *in vitro*.** (**A, B**) Representative Western blotting (A) and quantitative data (B) indicate the induction of N-Shh and Shh expression in MPC5 cells at different time points after Ang II treatment. Numbers (1 to 3) indicate each individual treatment in a given group. ^***^P < 0.001 versus control (n = 6). (**C, D**) Western blotting analyses demonstrate the expression of N-Shh and Shh in MPC5 cells treated with different concentrations of Ang II. Representative Western blot (C) and quantitative data (D) are presented. Numbers (1 to 3) indicate each individual culture plate in a given group. ^**^P < 0.01, ^***^P < 0.001 versus control (n = 6). (**E**) Experimental design. MPC5 cells were treated with Ang II (1 µM) for 6 h, and then washed to remove Ang II and continued to incubate for additional 48 h in serum-free medium. Conditioned media were collected and used to stimulate normal rat mesangial cells (HBZY-1) for 24 h. (**F**) Cell Counting Kit-8 (CCK8) assay shows the conditioned media from MPC5 cells treated with Ang II (Ang II-CM) stimulated HBZY-1 cell proliferation *in vitro*. ^***^P < 0.001 versus Ctrl-CM (n = 6). (**G, H**) Representative Western blotting (G) and quantitative data (H) show the expression of c-Myc and proliferating cell nuclear antigen (PCNA) in HBZY-1 cells after incubation with conditioned media. Numbers (1 to 3) indicate each individual treatment in a given group. ^***^P < 0.001 versus Ctrl-CM (n = 6). (**I, J**) Representative Western blotting (I) and quantitative data (J) show the expression of FN, α-smooth muscle actin (α-SMA), and plasminogen activator inhibitor-1 (PAI-1) in HBZY-1 cells after incubation with conditioned media. Numbers (1 to 3) indicate each individual treatment in a given group. ^**^P < 0.01, ^***^P < 0.001 versus Ctrl-CM (n = 6). (**K**, **L**) Representative micrographs (K) and quantitative data (L) show immunofluorescence staining of fibronectin (FN) in HBZY-1 cells after incubation with conditioned media from MPC5 cells. Arrows indicate positive staining. Scale bar, 25 µm. ^***^P < 0.001 versus Ctrl-CM (n = 3). (**M, N**) Representative Western blotting (M) and quantitative data (N) demonstrate the reduction of CD63 and TSG101 expression after DMA treatment. Numbers (1 to 3) indicate each individual culture plate in a given group. ^***^P < 0.001 versus controls; ^†††^*P* < 0.001 versus Ang II-CM treatment alone (n = 6). (**O, P**) Blockade of EV generation by DMA abolished mesangial cell activation induced by MPC5 conditioned media. Representative micrographs (O) and quantitative data (P) show FN expression in different groups as indicated. Arrows indicate positive staining. Scale bar, 25 µm. ^***^P < 0.001 versus controls; ^†††^*P* < 0.001 versus Ang II-CM treatment alone (n = 3). (**Q, R**) Western blot analyses show that blockade of EV secretion by DMA inhibited FN, PDGFR-β, and α-SMA expression in HBZY-1 cells induced by MPC5 conditioned media. Representative Western blot (Q) is shown. Numbers (1 to 3) indicate each individual culture in given group. Graphic presentation (R) indicates the relative protein levels of FN, PDGFR-β, and α-SMA. ^**^P < 0.01 versus controls, ^††^*P* < 0.01, ^†††^*P* < 0.001 versus Ang II-CM (n = 6). (**S, T**) Western blot analyses show that blockade of EV secretion by DMA inhibited c-Myc and PCNA expression in HBZY-1 induced by MPC5 conditioned media. Representative Western blot (S) is shown. Numbers (1 to 3) indicate each individual culture in given group. Graphic presentation (T) indicates the relative protein levels of c-Myc and PCNA. ^**^P < 0.01, ^***^P < 0.001 versus controls, ^††^*P* < 0.01, ^†††^*P* < 0.001 versus Ang II-CM (n = 6).

Since Shh was identified as a major constituent of podocyte-derived EVs (Figure 1H-L), we then investigated its expression in cultured podocytes after Ang II stimulation. As shown in Figure 2A-D, both full-length Shh precursor (45 kDa) and active N-terminal Shh (N-Shh, 19 kDa) were upregulated by Ang II in a time- and dose-dependent manner. To study the actions of podocyte-derived EVs enriched with Shh in mesangial cells, we carried out experiments using an in vitro system. MPC5 cells were initially treated with Ang II for 6 h, and then Ang II was removed, followed by incubation in serum-free medium for 24 h. Conditioned media were collected and used to stimulate rat mesangial cells (HBZY-1) (Figure 2E). We evaluated the potential of conditioned media from MPC5 cells treated with Ang II (Ang II-CM) to stimulate mesangial cell proliferation. The Cell Counting Kit-8 (CCK-8) assay revealed an increase in HBZY-1 cell mass after incubation with Ang II-CM (Figure 2F). Western blotting indicated the induction of c-Myc and proliferating cell nuclear antigen (PCNA) in the mesangial cells following incubation with Ang II-CM (Figure 2G and H).

We found that Ang II-CM also induced mesangial cell activation and promoted ECM production. As shown in Figure 2I-L, Ang II-CM induced the expression of fibronectin (FN), α-smooth muscle actin (α-SMA), and plasminogen activator inhibitor-1 (PAI-1), compared to controls (Ctrl-CM). To further investigate the role of podocyte-derived EVs in mediating mesangial activation, MPC5 cells were pretreated with dimethyl amiloride (DMA), a blocker of EVs biogenesis and release. Western blotting analyses of CD63 and TSG101 confirmed that DMA inhibits EVs secretion (Figure 2M and N). We found that blockade of EVs release by DMA inhibited the expression of FN, platelet-derived growth factor receptor β (PDGFR-β), α-SMA, c-Myc and PCNA (Figure 2O-2T). These results demonstrate that inhibition of EVs release from podocytes attenuates mesangial cell activation.

### Podocyte-derived EVs mediate mesangial cell activation and proliferation *in vitro*

**Figure 3:**
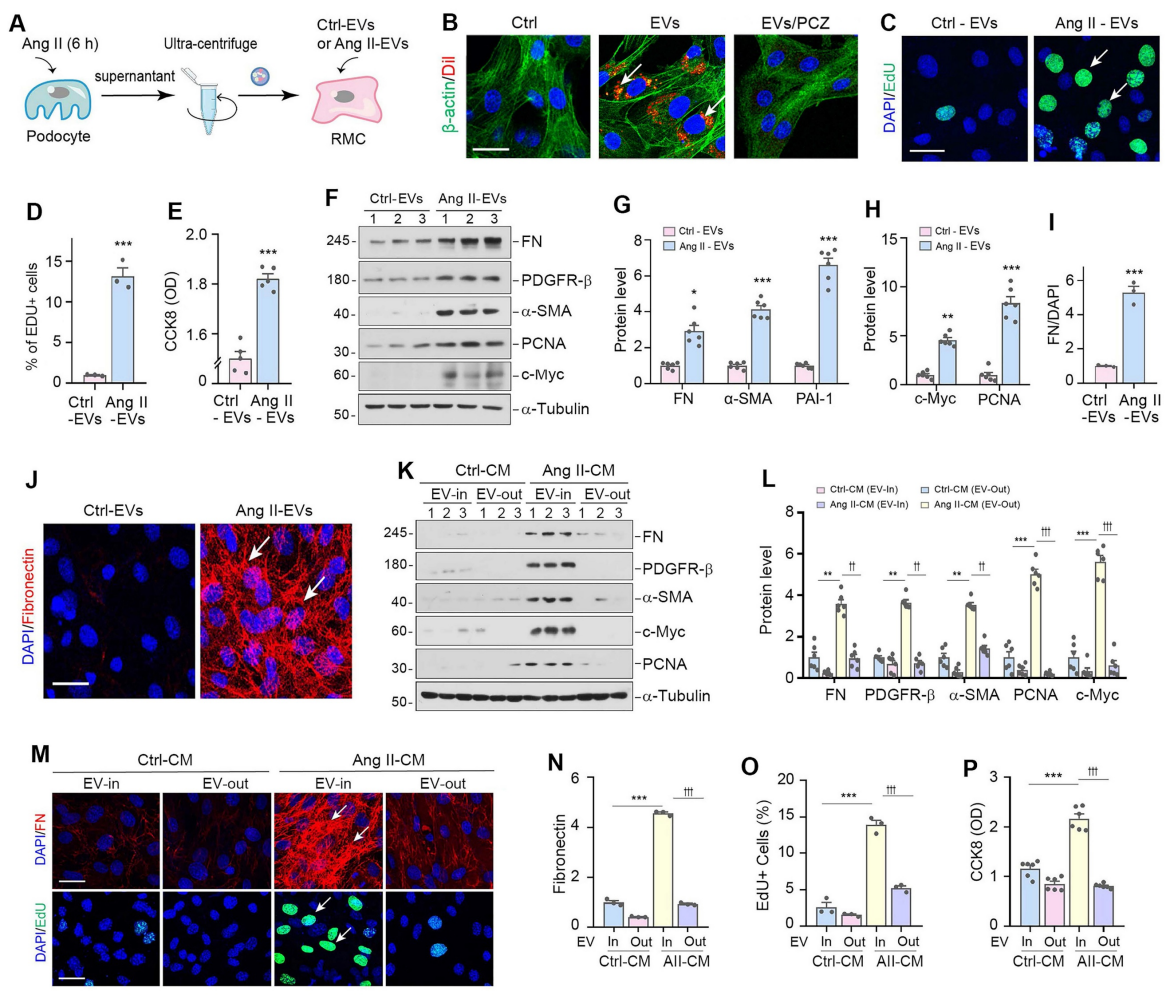
** Podocyte-derived EVs mediate mesangial cell activation and proliferation *in vitro*.** (**A**) Diagram shows the experimental design. EVs were isolated from conditioned media by ultracentrifugation. (**B**) Fluorescent staining confirms the intercellular transfer of MPC5 cell-derived EVs in HBZY-1 cells. MPC5 cells were incubated with Dil-C18 (red), a fluorescent lipophilic membrane dye for a long-term tracing. HBZY-1 cells were pretreated with or without the endocytosis inhibitor prochlorperazine (PCZ). MPC cell-derived EVs were then isolated and incubated with HBZY-1 cells for 24 h, followed by immunofluorescence staining for β-actin (green). Arrows indicate MPC5 cell-derived EVs. Scale bar, 25 µm. (**C-E**) EVs derived from Ang II-treated MPC5 cells stimulated HBZY-1 cell proliferation *in vitro*. EdU incorporation (C) and its quantitative data (D) are shown. Arrows indicate positive staining. Scale bar, 25 µm. ^***^P < 0.001 versus Ctrl-EVs (n = 3). Graphic presentation (E) indicates the Cell Counting Kit-8 assay. ^***^P < 0.001 versus Ctrl-EVs (n = 6). (**F-H**) Western blot analyses show protein expression of FN, PDGFR-β, α-SMA, PCNA and c-Myc in HBZY-1 cells exposed to Ctrl-EVs or Ang II-EVs. Representative Western blot (F) and quantitative data (G, H) are presented. Numbers (1 to 3) indicate each individual treatment in a given group. ^*^P < 0.05, ^**^P < 0.01, ^***^P < 0.001 versus Ctrl-EVs (n = 6). (**I**, **J**) Representative micrographs (J) and quantitative data (I) show FN-positive staining in HBZY-1 cells after incubation with EVs derived from MPC5 cells. Arrows indicate positive staining. Scale bar, 25 µm. ^***^P < 0.001 versus Ctrl-EVs (n = 3). (**K, L**) EVs are required for mediating HBZY-1 cell activation induced by MPC5 cells conditioned media. Western blot analyses show that FN, PDGFR-β, α-SMA, c-Myc and PCNA expression in different groups as indicated. Numbers (1 to 3) indicate each individual culture plate in a given group. Quantitative data (L) is presented. ^**^P < 0.01, ^***^P < 0.001 versus Ctrl-CM (EV-In); ^††^*P* < 0.01, ^†††^*P* < 0.001 versus Ang II-CM (EV-Out) (n = 6). (**M-O**) Representative micrographs (M) and quantitative data (N) show FN-positive staining in different groups as indicated. Arrows indicate positive staining. Scale bar, 25 µm. ^***^P < 0.001 versus Ctrl-CM, ^†††^*P* < 0.001 versus Ang II-CM (n = 3). Representative micrographs (M) and quantitative data (O) show EdU incorporation in different groups as indicated. Arrows indicate positive staining. Scale bar, 25 µm. ^***^P < 0.001 versus Ctrl-CM, ^†††^*P* < 0.001 versus Ang II-CM (n = 3). (**P**) Cell Counting Kit-8 assay shows MPC5 cells conditioned media lacking EVs failed to induce HBZY-1 cell proliferation. ^***^P < 0.001 versus Ctrl-CM, ^†††^*P* < 0.001 versus Ang II-CM (n = 6).

To directly examine the function of podocyte-derived EVs in mediating mesangial cell activation, we isolated EVs from the conditioned medium of cultured MPC5 cells treated with or without Ang II (Figure 3A). We then labelled the EVs from Ang II-treated MPC5 cells with Dil, a fluorescent lipophilic membrane dye, for long-term tracking. Figure 3B shows that Dil-labelled EVs were taken up by mesangial cells after incubation with HBZY-1 cells, as outlined by staining for the β-actin cytoskeleton. To elucidate the mechanism of EV uptake by mesangial cells, we pretreated mesangial cells with the endocytosis inhibitor prochlorperazine (PCZ) for 1 h. As shown in Figure 3B, inhibition of endocytosis by PCZ largely prevented the entry of Dil-labeled EVs into the mesangial cells, suggesting that podocyte-derived EVs are primarily uptaken by the recipient cells through endocytosis.

We subsequently evaluated the capacity of Ang II-EVs to stimulate HBZY-1 cell activation and proliferation. Figure 3C-E showed that Ang II-EVs increased the 5-ethynyl-2'-deoxyuridine (EdU)-positive cells and enlarged mesangial cell population by CCK-8 assay. Similarly, Ang II-EVs also induced the expression of FN, PDGFR-β, α-SMA, PCNA and c-Myc in HBZY-1 cells (Figure 3F-J). To test whether EVs are necessary for mediating podocyte-mesangial communication, we incubated HBZY-1 cells with complete conditioned media (EV In) or EV-depleted conditioned media (EV Out). As demonstrated in Figure 3K and L, complete Ang II-CM induced FN, PDGFR-β, α-SMA, c-Myc, and PCNA expression in HBZY-1 cells. Depletion of EV from Ang II-CM (EV Out) abolished the induction of these proteins. Consistently, FN and EdU staining, as well as CCK-8 assay, produced similar results (Figure 3M-P).

### Shh in podocyte-derived EVs mediate podocyte-mesangial communication

**Figure 4:**
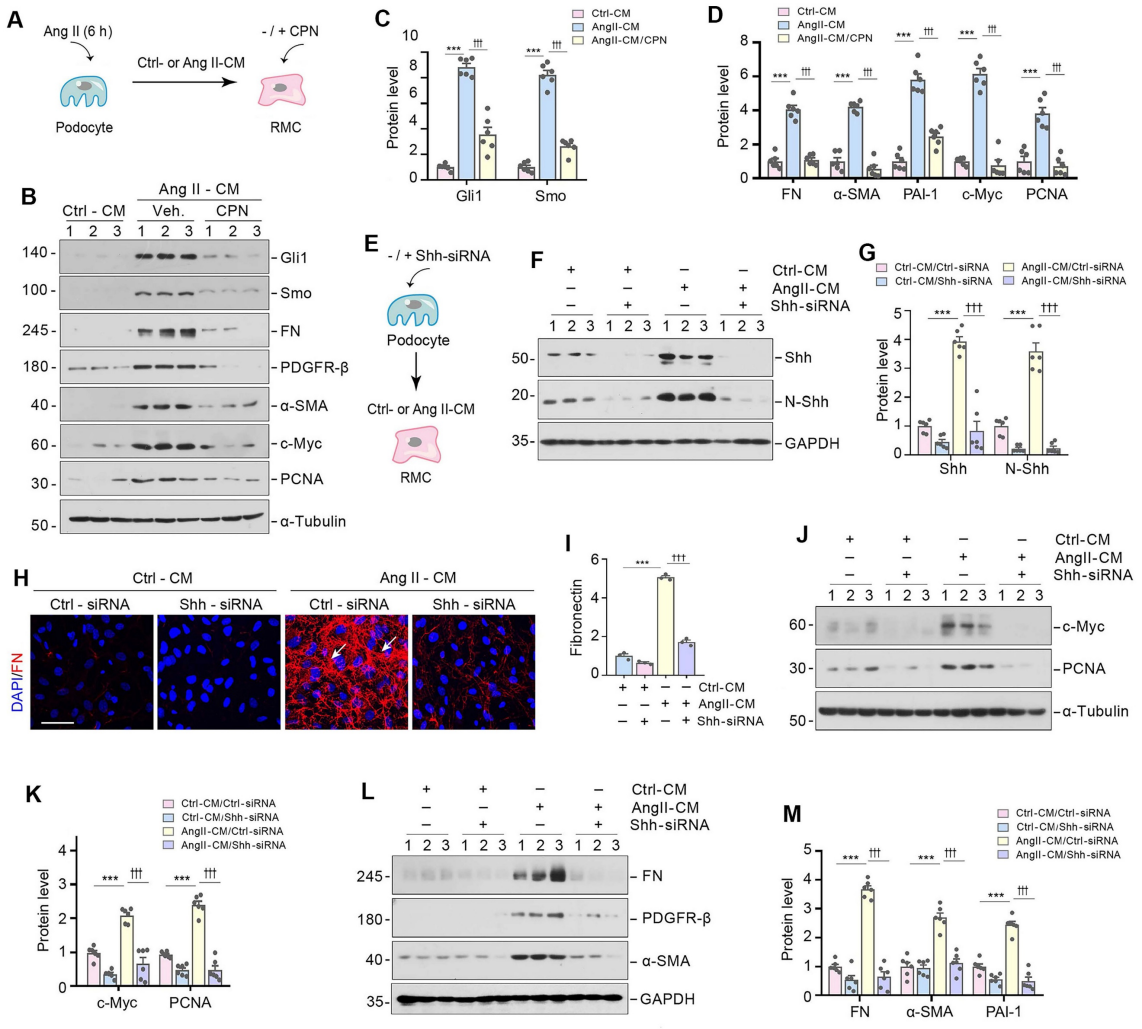
** Shh in podocyte-derived EVs mediate podocyte-mesangial communication.** (**A**) Experimental design show Shh signal are inhibited in HBZY-1 cells prior to stimulation with MPC5 cells conditioned media. HBZY-1 cells were treated with CPN for 1 h, and then treated with Ctrl-CM or Ang II-CM for 24 h. (**B-D**) Western blot (B) and quantitative data (C, D) show the upregulation of Gli1, Smo, FN, PDGFR-β, α-SMA, c-Myc and PCNA expression after incubation with Ang II-CM, but these effects are abolished by CPN treatment. Numbers (1 to 3) indicate each individual treatment in a given group. ^***^P < 0.001 versus Ctrl-CM, ^†††^*P* < 0.001 versus Ang II-CM (n = 6). (**E**) Experimental design showing knockdown of Shh in MPC5 cells prior to collection of conditioned media. (**F, G**) Western blot (F) and quantitative data (G) showing protein levels of Shh and N-Shh in different groups as indicated. Numbers (1 to 3) indicate each individual treatment in a given group. ^***^P < 0.001, ^†††^*P* < 0.001 (n = 6). (**H, I**) Representative micrographs (H) and quantitative data (I) show Knockdown of Shh in MPC5 cells reduced FN-positive staining in HBZY-1 cells after incubation with conditioned media. Arrows indicate positive staining. Scale bar, 50 µm. ^***^P < 0.001, ^†††^*P* < 0.001 (n = 6). (**J, K**) Western blot analyses show protein expression of c-Myc and PCNA in different groups of HBZY-1 cells as indicated. Representative Western blot (J) and quantitative data (K) are presented. Numbers (1 to 3) indicate each individual treatment in a given group. ^***^P < 0.001, ^†††^*P* < 0.001 (n = 6). (**L, M**) Western blot analyses show protein expression of FN, PDGFR-β and α-SMA in different groups of HBZY-1 cells as indicated. Representative Western blot (L) and quantitative data (M) are presented. Numbers (1 to 3) indicate each individual treatment in a given group. ^***^P < 0.001, ^†††^*P* < 0.001 (n = 6).

We next investigated whether Shh in the podocyte-derived EVs is responsible for mediating mesangial cell activation and proliferation. To this end, HBZY-1 cells were pre-incubated for 30 min with cyclopamine (CPN), a small molecule inhibitor of hedgehog signaling via repressing Smo, followed by incubation with Ang II-CM for 24 h (Figure 4A). As shown in Figure 4B-D, CPN inhibited the expression of Smo and its downstream molecule Gli1 and largely abolished the induction of FN, PDGFR-β, α-SMA, c-Myc, and PCNA expression caused by Ang II-CM. In addition, we inhibited Shh signaling in MPC5 cells by transfecting them with Shh-specific siRNA (Shh-siRNA) or scrambled siRNA (Ctrl-siRNA) (Figure 4E-G). Immunostaining confirmed that the knockdown of Shh abolished the FN expression induced by Ang II-CM (Figure 4H and I). Similarly, knockdown of Shh also prevented Ang II-CM from inducing c-Myc, PCNA, FN, PDGFR-β, and α-SMA expression in HBZY-1 cells (Figure 4J-M). Therefore, these results illustrate that Shh in the podocyte-derived EVs appears responsible for mediating mesangial cell activation.

### The production of EVs and Shh is increased in glomerular disease model

**Figure 5:**
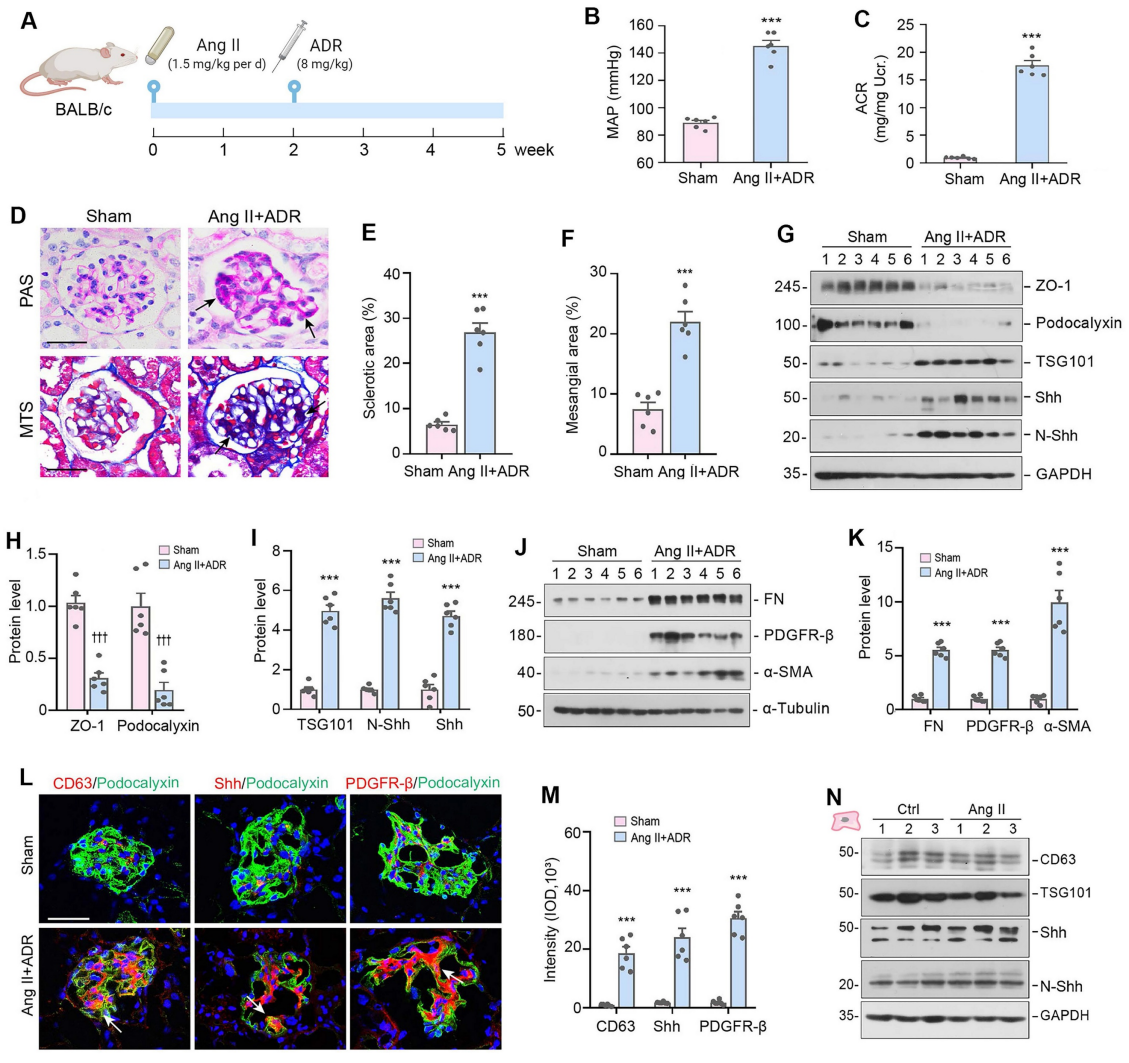
** The production of EVs and Shh is increased in mouse model of glomerulosclerosis.** (**A**) Diagram shows the experimental design. The time points when osmotic pump was placed into the subcutaneous space of mice and intravenous injection of ADR at the dose of 8 mg/ kg body weight are indicated, respectively. Mice were infused with Ang II at 1.5 mg/ kg per day with osmotic pumps. (**B, C**) Graphic presentations show the mean arterial pressure (MAP) levels (B) and urinary albumin-to creatinine ratio (ACR) levels (C) in Ang II/ADR mice. ^***^P < 0.001 versus sham controls (n = 6). (**D-F**) Representative micrographs (D) and quantifications show the fraction of collagen deposition (E) and mesangial area (F) in Ang II/ADR mice. Paraffin sections were subjected to periodic acid-Schiff (PAS) staining (upper) and Masson's trichrome staining (bottom), respectively. Arrow indicates positive staining. Scale bar, 50 µm. ^***^P < 0.001 versus sham controls (n = 6). (**G-I**) Representative Western blotting (G) and quantitative data show the loss of ZO-1 and podocalyxin (H) and the induction of TSG101, N-Shh and Shh (I) in the glomeruli of Ang II/ADR mice. Numbers (1 to 6) indicate each individual animal in a given group. ^***^P < 0.001, ^†††^*P* < 0.001 versus sham controls (n = 6). (**J, K**) Western blotting analyses demonstrate the expression of FN, PDGFR-β and α-SMA in the glomeruli of Ang II/ADR mice. Representative Western blot (J) and quantitative data (K) are presented. Numbers (1 to 6) indicate each individual animal in a given group. ^***^P < 0.001 versus sham controls (n = 6). (**L, M**) Representative micrographs and quantifications of double immunofluorescence staining show CD63 (left), Shh (middle) and PDGFR-β (right) expression in the glomeruli of Ang II/ADR mice. Kidney sections were co-stained for podocalyxin (green) and CD63, Shh or PDGFR-β (Red), respectively. Scale bar, 50 µm. ^***^P < 0.001 versus controls (n = 6). (**N**) Representative Western blotting of CD63, TSG101, N-Shh and Shh in HBZY-1 cells after incubation with Ang II for 24 h are shown. Numbers (1 to 3) indicate each individual culture in a given group.

We then investigated the production of EVs and Shh expression in mouse model of glomerular diseases in vivo. To achieve robust podocyte injury and glomerulosclerotic lesions, we employed a mouse model of glomerular disease induced by a combined Ang II and ADR injuries (Figure 5A). Specifically, male BALB/c mice were infused with Ang II at a rate of 1.5 mg/kg per day using osmotic pumps. After 14 days, the mice were injected with ADR at a dose of 8 mg/kg body weight. The mice were euthanized 5 weeks after Ang II infusion (Figure 5A). As demonstrated in Figure 5B and C, mean arterial pressure (MAP) levels increased and heavy proteinuria developed in this model. Furthermore, periodic acid-Schiff (PAS) and Masson's trichrome staining showed mesangial expansion and glomerulosclerosis in these Ang II-infused and ADR-injected mice (Figure 5D-F).

Podocyte injury was evident in this model, as there was significant loss of ZO-1 and podocalyxin (Figure 5G and H). Interestingly, the loss of ZO-1 and podocalyxin was closely associated with the induction of TSG101, N-Shh and Shh (Figure 5G and I). Glomerular induction of FN, PDGFR-β, and α-SMA was also observed at 5 weeks after Ang II infusion, which was shown by Western blot analyses of glomerular lysates isolated from the whole kidney (Figure 5G-K). Immunofluorescence staining also gave rise to similar results (Figure 5L and 5M). Notably, immunostaining showed a marked increase of CD63, Shh and PDGFR-β in the mesangial areas of the glomeruli. However, there was little or no difference in the expression of CD63, TSG101, N-Shh and Shh between control and Ang II-treated HBZY-1 cells (Figure 5N). These findings suggest that the increased CD63 and Shh in mesangial areas were not produced by mesangial cells *in situ* but transferred from podocytes.

### Podocyte-derived EVs promote glomerulosclerosis *in vivo*

**Figure 6:**
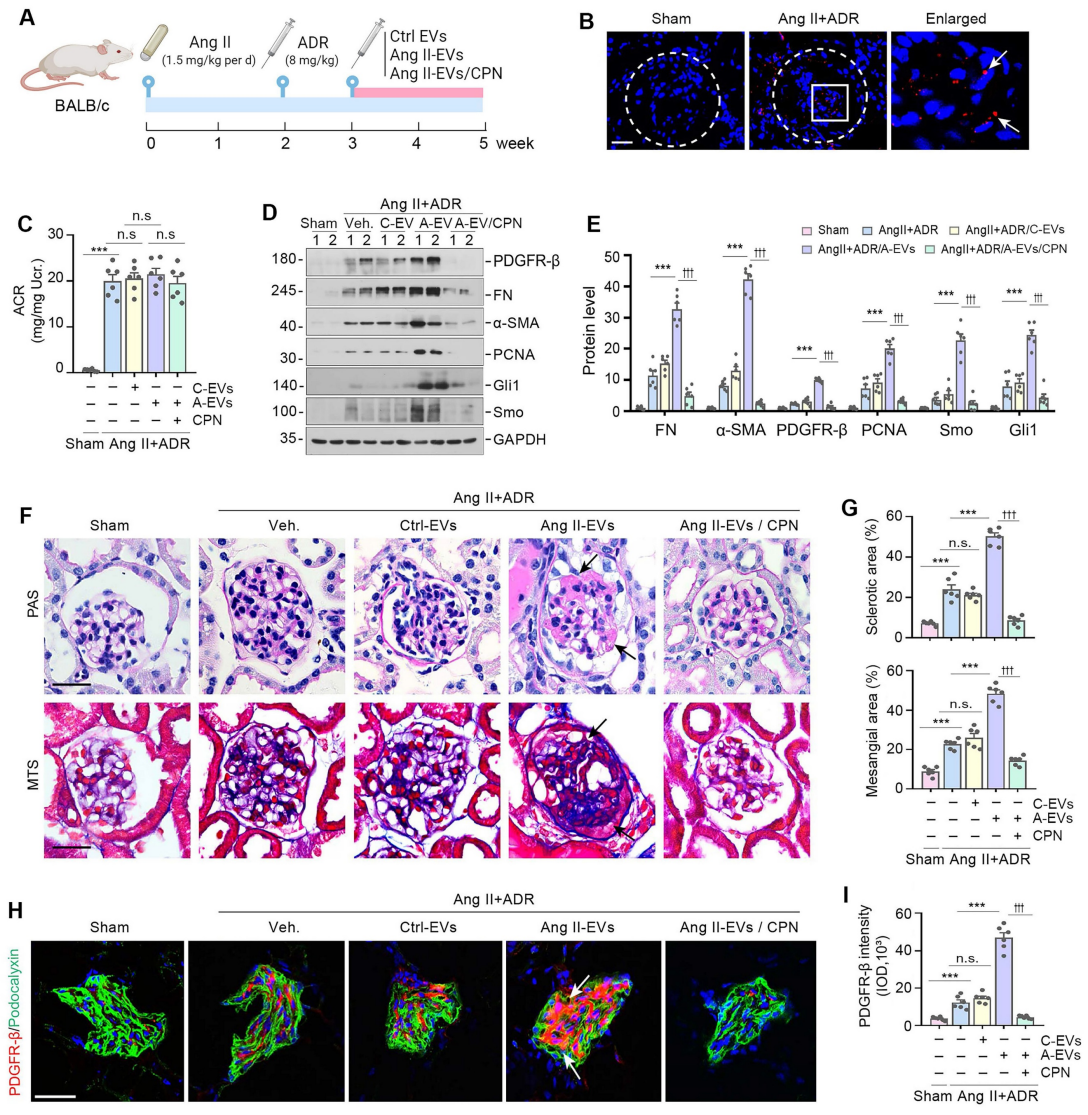
** Podocyte-derived EVs promote glomerulosclerosis *in vivo*.** (**A**) Diagram shows the experimental design. The time points of the osmotic pump of Ang II being placed into the subcutaneous space of mice (at the dose of 1.5 mg/kg body weight per day), the intravenous injection of ADR (8 mg/kg body weight), the intravenous injection of MPC cells-derived EVs (Ctrl-EVs or Ang II-EVs, 1 mg per mouse per time point, once per two days) and the CPN treatment (5 mg/kg body weight), are indicated. (**B**) Imaging of fluorescence intensity showed Dil-C18-labeled EVs in the glomeruli at 12 h after the last intravenous injection. EVs isolated from Ang II-treated MPC5 cells were labelled with fluorescent lipophilic membrane dye Dil-C18, and then injected through the tail vein into Sham (left) and Ang II/ADR mice (right). Part of the glomerulus was enlarged and positive staining indicated by arrows. (**C**) Graphic presentation shows the urinary albumin-to creatinine ratio (ACR) levels in different groups after Ang II and ADR treatment. ^***^P < 0.001, n.s., not significant (n = 6). (**D, E**) Western blot analyses show expression of PDGFR-β, FN, α-SMA, PCNA, Gli1 and Smo in different groups of glomeruli as indicated. Numbers (1 to 2) indicate each individual animal in a given group. Quantitative data is presented (E). ^***^P < 0.001 versus Ang II+ADR mice; ^†††^*P* < 0.001 versus CPN treated with Ang II-EVs (n = 6). (**F, G**) Representative micrographs (F) and quantifications (G) show the collagen deposition and mesangial area in different groups as indicated. Paraffin sections were subjected to periodic acid-Schiff (PAS) staining (upper) and Masson's trichrome staining (bottom), respectively. Arrows indicate positive staining. Scale bar, 50 µm. ^***^P < 0.001, ^†††^*P* < 0.001, n.s., not significant (n = 6). (**H, I**) Representative micrographs and quantification of double immunofluorescence staining show PDGFR-β expression in different groups of glomeruli as indicated. Kidney sections were co-stained for PDGFR-β (Red) and podocalyxin (green), respectively. Arrows indicate positive staining. Scale bar, 50 µm. ^**^P < 0.01, ^***^P < 0.001, ^†††^*P* < 0.001, n.s., not significant (n = 6).

To investigate the relevance of podocyte-derived EVs to glomerulosclerosis in vivo, we carried out animal studies using Ang II/ADR model by injection of the Ang II-EVs isolated from Ang II-treated MPC5 cells, as shown in Figure 6A. Ang II/ADR mice were given same amounts of Ctrl-EVs or Ang II-EVs every two days from the third week after Ang II infusion (Figure 6A). To inhibit hedgehog signaling, we performed daily injections of CPN at a dose of 5 mg/kg body weight through the tail vein. First, we examined whether EVs could home in the glomeruli of the kidney after tail vein injection. As shown in Figure 6B, Dil-labeled EVs were detected in the glomeruli of diseased kidneys in Ang II/ADR injected mice, while they were much less abundant in the kidneys of healthy mice. Neither the injection of Ang II-EVs nor inhibition of Shh by CPN had any effect on the severity of albuminuria in Ang II/ADR mice (Figure 6C), indicating that podocyte-derived EVs or Shh do not affect podocyte function.

We then examined the effects of various treatments on glomerulosclerosis in these Ang II/ADR mice. As shown in Figure 6D and E, Ang II-EVs significantly upregulated the expression of PDGFR-β, FN, α-SMA, PCNA, Gli1 and Smo in the Ang II/ADR mice. However, the induction of these proteins was negated by inhibiting Shh with CPN. Consistently, PAS and Masson's trichrome staining showed that Ang II-EVs aggravated glomerulosclerotic lesions in Ang II/ADR mice, compared with Ctrl-EVs. However, treatment with CPN largely abolished the effects of Ang II-EVs (Figure 6F and G). Similar results were obtained when PDGFR-β was assessed by immunostaining (Figure 6H and I). Together, these findings indicate that EVs-packaged Shh derived from podocytes has a significant role in promoting mesangial activation and glomerulosclerosis in vivo.

### Blockade of EVs secretion ameliorates glomerulosclerosis *in vivo*

**Figure 7:**
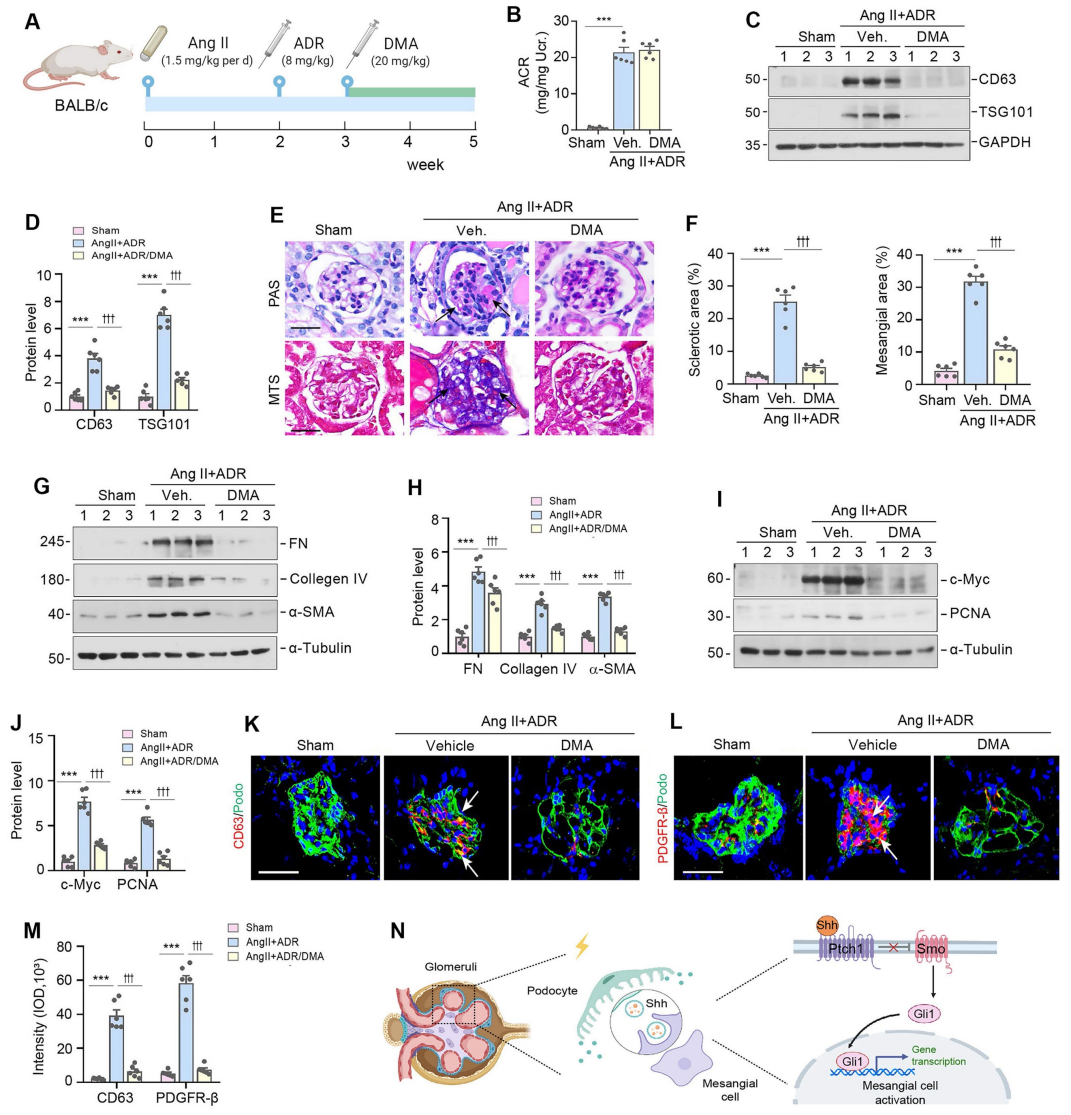
** Blockade of EV secretion ameliorates glomerulosclerosis *in vivo*.** (**A**) Diagram shows the experimental design. The time points of the osmotic pump of Ang II being placed into the subcutaneous space of mice (at the dose of 1.5 mg/kg body weight per day), the intravenous injection of ADR (8 mg/kg body weight) and the daily intraperitoneal injection of dimethyl amiloride DMA (20 mg/kg body weight in 0.9% saline), are indicated. (**B**) DMA treatment did not affect albuminuria in Ang II/ADR mice. Urinary albumin-to creatinine ratio (ACR) levels were shown. ^***^P < 0.001, n.s., not significant (n = 6). (**C, D**) DMA inhibits EVs secretion of glomeruli in Ang II/ADR mice. Representative Western blot (C) and quantitative data (D) show the reduction of glomerular CD63 and TSG101 expression after DMA treatment. Numbers (1 to 3) indicate each individual animal in a given group. ^***^P < 0.001, ^†††^*P* < 0.001 (n = 6). (**E-F**) Representative micrographs (E) and quantifications (F) show the collagen deposition and mesangial area in different groups as indicated. Paraffin sections were subjected to periodic acid-Schiff (PAS) staining (upper) and Masson's trichrome staining (bottom), respectively. Arrows indicate positive staining. Scale bar, 50 µm. ^***^P < 0.001, ^†††^ *P* < 0.001 (n = 6). (**G, H**) Western blot analyses show that DMA inhibited glomerular expression of mesangial activation-related proteins in Ang II/ADR mice. Representative Western blot (G) and quantitative data (H) are shown. Numbers (1 to 3) indicate each individual animal in a given group. ^***^P < 0.001, ^†††^*P* < 0.001 (n = 6). (**I, J**) Representative Western blot (I) and quantitative data (J) show the protein levels of c-Myc and PCNA in different groups of glomeruli as indicated. Numbers (1 to 3) indicate each individual animal in a given group. ^***^P < 0.001, ^†††^*P* < 0.001 (n = 6). (**K-M**) Representative micrographs (K, L) and quantification (M) of double immunofluorescence staining show CD63 (K) and PDGFR-β (L) expression of glomeruli in different groups as indicated. Kidney sections were co-stained for podocalyxin (green), and CD63 or PDGFR-β (Red), respectively. Arrows indicate positive staining. Scale bar, 50 µm. ^***^P < 0.001, ^†††^*P* < 0.001 (n = 6). (**N**) Schematic diagram illustrating the role of podocyte-derived EVs enriched with sonic hedgehog (Shh) in promoting mesangial cell activation and glomerulosclerosis. Following podocyte injury, these EVs facilitate the delivery of Shh to mesangial cells, thereby activating them via the Shh/Smoothened/Gli1 signaling pathway, which ultimately promotes mesangial activation and glomerulosclerosis.

To further confirm the role of EVs in glomerulosclerosis, we used DMA to block the release of EVs in Ang II/ADR mice. The experimental design is presented in Figure 7A. DMA treatment did not affect albuminuria (Figure 7B), but blocked the biogenesis and secretion of EVs, as illustrated by a reduced expression of CD63 and TSG101 (Figure 7C-D). Interestingly, we found that blockade of EVs biogenesis and secretion by DMA markedly alleviated glomerulosclerotic lesions, as shown by PAS and Masson's trichrome staining (MTS) (Figure 7E-F). Similar results were obtained by Western blotting analyses of multiple mesangial cell activation and glomerulosclerosis-related proteins including FN, collagen IV, α-SMA, c-Myc, and PCNA (Figure 7G-J). Consistently, immunofluorescence staining also showed that DMA abolished the upregulation of CD63 and PDGFR-β in the injured glomeruli of Ang II/ADR mice (Figure 7K-M). Taken together, these results indicate that EVs derived from damaged podocytes play a crucial role in promoting mesangial cell activation and glomerulosclerosis by delivering Shh to mesangial cells (Figure 7N).

## Discussion

Glomerular disease is typically characterized by proteinuria and glomerulosclerosis, which are caused by podocyte injury and mesangial cell activation, respectively 31, 32. In this study, we investigated the role of podocyte-derived EVs in mediating communication between podocytes and mesangial cells. We demonstrate that podocytes transmit Shh signals via EVs, which in turn promote mesangial cell activation and glomerulosclerosis. Moreover, blockade of EV biogenesis and secretion by DMA, or inhibition of Shh signaling or expression through pharmacological or genetic approaches, suppresses mesangial cell activation. Similarly, inhibition of EV biogenesis and secretion or Shh signaling by CPN ameliorates glomerulosclerosis in vivo. These results highlight that EVs serve as potent mediators of the signal exchange between podocytes and mesangial cells. Our findings offer novel insights into the mechanisms on how podocyte injury and proteinuria cause mesangial cell activation and glomerulosclerosis in CKD.

Podocytes are unique and specialized cells that form part of the glomerular filtration barrier, and their injury often leads to impaired glomerular filtration, eventually resulting in proteinuria 33, 34. It is well known that podocyte injury leading to proteinuria is typically an early event of glomerular disease, while mesangial cell activation and proliferation leading to glomerulosclerosis is the hallmark of a later and often irreversible event in glomerulopathy. However, not all proteinuric glomerular diseases progress to glomerulosclerosis or fibrotic CKD. This discrepancy raises questions about whether proteinuria should be considered a reliable surrogate marker for CKD progression, as it does not consistently predict the decline in estimated glomerular filtration rate (eGFR) or the extent of tissue fibrosis 35-38. Currently, little is known about the intimate connection mechanisms between podocyte injury and mesangial cell activation, the initial step leading to glomerulosclerosis 39, 40. Earlier studies have shown that Shh can be a potential mediator linking podocyte dysfunction to mesangial cell activation 41. However, how Shh, a lipid-modified protein, reaches to mesangial cells remains mysterious. It is plausible that as a lipid-modified protein, Shh may readily fuse with the lipid membranes of EVs, enabling its delivery to recipient cells 42, 43. We herein corroborate that the principal components of Shh signal cascade including Shh, N-Shh, Smo and Gli1 are all encapsulated into podocyte-derived EVs, which can then fuse with the cell membrane, releasing their contents into the mesangial cell (Figure 1). Although EVs carry a variety of cargoes including proteins, mRNA, microRNA, lipids and metabolites, we demonstrate that Shh signal probably plays a major role in mediating podocyte-mesangial cell communication, as inhibition of Shh signaling by CPN or knockdown of Shh expression in podocytes abolishes mesangial cell activation (Figures 4 and 6). In view of that podocyte-derived EVs contain a wide array of proteins involved in Shh signaling such as N-Shh, Smo and Gli1, it is reasonable to speculate that EVs-mediated podocyte-mesangial cell communication is both efficient and robust.

Using electron microscopy and immunofluorescence staining, we observed abundant EVs in the glomeruli of FSGS patients, located between podocytes and the mesangial area. Co-localization of Shh and the exosomal marker CD63 in the glomeruli further suggests that EVs carry Shh in the pathogenesis of glomerular lesions (Figure 1), underscoring the critical role of EVs in the pathogenesis of proteinuric CKD. Consistent with this notion, an increased expression of EV markers (TSG101 and CD63), podocyte marker (nephrin) and Shh/N-Shh is also detected in the urine of CKD patients (Figure 1). Damaged podocytes encapsulate large amounts of Shh, Smo and Gli1 into EVs, underscoring that EVs serve not only as vehicles for cellular communication but also as active carriers of Shh signalosome (Figure 1). Interestingly, although mesangial cells did not produce more EVs or Shh in response to Ang II stimulation in vitro (Figure 5N), remarkable accumulation of exosomal marker CD63 and Shh is predominantly localized in the glomerular mesangium of the diseased kidneys induced by Ang II and ADR (Figure 5L), suggesting that podocyte-derived EVs are precisely targeted to mesangial cells, leading to their activation, proliferation and matrix production in vivo. Furthermore, blockade of the EVs biogenesis and release by DMA alleviates mesangial cell activation and glomerulosclerosis in vivo (Figure 7). Notably, Ang II-CM lacking EVs (EV-out) failed to stimulate mesangial cell activation (Figure 3), indicating that it is the EVs, rather than soluble factors, that mediate podocyte-mesangial communication in diseased kidneys. These findings accentuate that EVs are essential mediators of podocyte-mesangial communication, and inhibiting their biogenesis or release may decouple podocyte injury/proteinuria from mesangial cells activation/glomerulosclerosis, thereby providing a novel therapeutic approach for glomerulosclerosis in CKD.

Another intriguing finding in this study is that Shh is selectively sorted into EVs following podocyte injury (Figure 1), which is then secreted to the neighboring mesangium unidirectionally. In such way, EVs act as effective vehicles for transporting Shh from podocytes to mesangial cells. This notion is supported by several lines of evidence, including the upregulation of Shh and N-Shh in the EVs isolated from damaged podocytes, and the mitigated glomerulosclerosis by Shh signaling inhibitor CPN (Figure 6). Moreover, Ang II-induced podocyte injury leads to co-expression of Shh and CD63, confirming the presence of Shh in EVs (Figure 1). These EVs are readily taken up by mesangial cells, largely through a mechanism of endocytosis (Figure 3B), triggering mesangial activation in vitro and exacerbating glomerulosclerosis in vivo (Figures 3 and 6). These results provide solid evidence that Shh is transmitted from podocytes to mesangial cells via EVs, contributing to mesangial cell activation and glomerulosclerosis. However, the present study still lacks of direct evidence to prove that podocyte-derived EVs are actually taken up by mesangial cells in vivo, although it is attested in vitro (Figure 3B). In this regard, labeling and tracing podocyte-derived EVs in vivo might serve as effective approaches and should be explored in future investigations. Clearly, more studies are needed in this area.

As the podocyte-derived Shh-enriched EVs are increased in the urine of proteinuric CKD patients (Figure 1), it is conceivable that these EVs may affect tubular epithelial cells, leading to subsequent tubulointerstitial lesions. However, earlier studies show that Shh does not directly induce cell proliferation in human proximal tubular (HKC-8) or murine collecting duct (mIMCD-3) cells 44, 45, suggesting that mesangial cells are probably the primary targets of podocyte-derived EVs in glomerular disease. Nevertheless, we cannot exclude the possibility that the Shh-enriched EVs inside tubular lumen may traverse across renal interstitial space to regulate fibroblast activation.

In summary, we demonstrate herein a crucial role of EV-mediated intercellular communication in linking podocyte injury to mesangial cell activation. We show that glomerular injury increases EVs production and release from podocytes, which contain key components of Shh signaling and promote mesangial cell activation and the development of glomerulosclerosis. This study offers new insights into intercellular communication in the injured/stressed glomerulus and provides compelling evidence for coupling proteinuria to glomerulosclerosis. By targeting EV biogenesis and secretion, it may be feasible to decouple podocyte injury from glomerulosclerosis, presenting a promising therapeutic strategy for CKD treatment.

## Supplementary Material

Supplementary figures and tables.

## Abbreviations

ADR: Adriamycin
Ang II: angiotensin II
Ang II-CM: angiotensin II-treated conditioned media
Ang II-EVs: extracellular vesicles isolated from angiotensin II-treated conditioned media
α-SMA: α-smooth muscle actin
CCK-8: cell counting kit-8
CKD: chronic kidney disease
CPN: cyclopamine
Ctrl-CM: control-treated conditioned media
Ctrl-EVs: extracellular vesicles isolated from control-treated conditioned media
Ctrl-siRNA: scrambled siRNA
DMA: dimethyl amiloride
ECM: extracellular matrix
EdU: 5-ethynyl-2'-deoxyuridine
EV: extracellular vesicle
EV In: extracellular vesicle-complete conditioned media
EV Out: extracellular vesicle-depleted conditioned media
FN: fibronectin
Gli1: glioma-associated oncogene homolog 1
MAP: mean arterial pressure
MPC: mouse podocytes
MTS: masson's trichrome staining
N-Shh: N-terminal sonic hedgehog
NTA: nanoparticle tracking analysis
PAS: periodic acid-schiff
PCNA: proliferating cell nuclear antigen
PCZ: prochlorperazine
PDGFR-β: platelet-derived growth factor receptor β
PAI-1: plasminogen activator inhibitor-1
Shh: sonic hedgehog
Shh-siRNA: Shh-specific siRNA
Smo: smoothened
TEM: transmission electron microscopy
TSG101: tumor susceptibility gene 101
