# Supplementary material for: Extracellular vesicles play a central role in linking podocyte injury to mesangial activation in glomerular disease
Source: Theranostics. 2025 Apr 9;15(11):5121–37. doi: 10.7150/thno.110034 (PMC12036872; doi:10.7150/thno.110034)
Supplement: Supplementary file 1 — Supplementary figures and tables. [file thnov15p5121s1.pdf]

Supplementary Table S1. The sources of antibodies used in this study

Antibodies	Catalogue number	Company	Location
**Primary antibodies**			
anti-Fibronectin	F3648	Sigma-Aldrich	St. Louis, MO
anti-α-SMA	A2547	Sigma-Aldrich	St. Louis, MO
anti-α-SMA	Ab5694	Abcam	Cambridge, MA
anti-PAI-1	AF3828	R & D Systems	Minneapolis, MN
anti-PCNA	Sc-56	Santa Cruz Biotechnolog	Santa Cruz, CA
anti-CD63	Ab59479	Abcam	Cambridge, MA
anti-CD63	Ab217345	Abcam	Cambridge, MA
anti-TSG101	Ab83	Abcam	Cambridge, MA
anti-PDGFR-β	sc-432	Santa Cruz Biotechnology	Santa Cruz, CA
anti-Shh	Sc-9024	Santa Cruz Biotechnology	Santa Cruz, CA
anti-Gli1	Ab49314	Abcam	Cambridge, MA
anti-smoothened	Ab72130	Abcam	Cambridge, MA
anti-c-Myc	#5605S	Cell Signaling Technology	Danvers, MA
anti-collagen IV	Ab211228	Abcam	Cambridge, MA
anti-podocalyxin	AF1556	R & D Systems	Minneapolis, MN
anti-ZO-1	QF215185	Life Technologies	Carlsbad, CA
anti-α-tubulin	RM2007	Ray Antibody Biotech	Beijing, China
anti-GAPDH	RM2002	Ray Antibody Biotech	Beijing, China
anti-β-actin	RM2001	Ray Antibody Biotech	Beijing, China
**Secondary antibodies**			
Goat anti-mouse	BA1050	Boster Biological Technology	Wuhan, China
Goat anti-rabbit	BA1054	Boster Biological Technology	Wuhan, China
Rabbit anti-goat	BA1060	Boster Biological Technology	Wuhan, China
Donkey Anti-Mouse	715-065-150	Jackson ImmunoResearch	West Grove, PA
Donkey Anti-Rabbit	711-065-152	Jackson ImmunoResearch	West Grove, PA
Donkey Anti-Mouse	715-225-150	Jackson ImmunoResearch	West Grove, PA
Donkey Anti-Rabbit	711-165-152	Jackson ImmunoResearch	West Grove, PA
Donkey Anti-Goat	705-545-147	Jackson ImmunoResearch	West Grove, PA

Supplementary Table S2. Clinical data of CKD patients.

Characteristics	CKD patients
**Age at entry-years**	
Mean ± SEM	46.4±15.7
Range	20-57
**Scr (μmol/l)**	447.6±252.1
**eGFR (ml/min/1.73m^2)**	16.8±8.5
**UTP (g/24 h)**	5.6±3.4
**Diagnosis No.**	
FSGS	2
CKD Stage 5	1
Proliferative Sclerosing IgAN	1
DN	1
